# Deep transformer-based personalized dosimetry from SPECT/CT images: a hybrid approach for [^177^Lu]Lu-DOTATATE radiopharmaceutical therapy

**DOI:** 10.1007/s00259-024-06618-9

**Published:** 2024-01-25

**Authors:** Zahra Mansouri, Yazdan Salimi, Azadeh Akhavanallaf, Isaac Shiri, Eliluane Pirazzo Andrade Teixeira, Xinchi Hou, Jean-Mathieu Beauregard, Arman Rahmim, Habib Zaidi

**Affiliations:** 1 Division of Nuclear Medicine and Molecular Imaging, Department of Medical Imaging, Geneva University Hospital, CH-1211 Geneva, Switzerland; 2 Department of Radiology, University of British Columbia, Vancouver, BC Canada; 3 Cancer Research Centre and Department of Radiology and Nuclear Medicine, Université Laval, Quebec City, QC Canada; 4 Department of Nuclear Medicine, University Medical Center Groningen, University of Groningen, 9700 RB Groningen, Netherlands; 5 Department of Nuclear Medicine, University of Southern Denmark, DK-500 Odense, Denmark; 6 University Research and Innovation Center, Óbuda University, Budapest, Hungary

**Keywords:** Radiation dosimetry, Radionuclide therapy, Deep learning, Monte Carlo simulation, [^177^Lu]Lu-DOTATATE

## Abstract

**Purpose:**

Accurate dosimetry is critical for ensuring the safety and efficacy of radiopharmaceutical therapies. In current clinical dosimetry practice, MIRD formalisms are widely employed. However, with the rapid advancement of deep learning (DL) algorithms, there has been an increasing interest in leveraging the calculation speed and automation capabilities for different tasks. We aimed to develop a hybrid transformer-based deep learning (DL) model that incorporates a multiple voxel *S*-value (MSV) approach for voxel-level dosimetry in [^177^Lu]Lu-DOTATATE therapy. The goal was to enhance the performance of the model to achieve accuracy levels closely aligned with Monte Carlo (MC) simulations, considered as the standard of reference. We extended our analysis to include MIRD formalisms (SSV and MSV), thereby conducting a comprehensive dosimetry study.

**Methods:**

We used a dataset consisting of 22 patients undergoing up to 4 cycles of [^177^Lu]Lu-DOTATATE therapy. MC simulations were used to generate reference absorbed dose maps. In addition, MIRD formalism approaches, namely, single *S*-value (SSV) and MSV techniques, were performed. A UNEt TRansformer (UNETR) DL architecture was trained using five-fold cross-validation to generate MC-based dose maps. Co-registered CT images were fed into the network as input, whereas the difference between MC and MSV (MC-MSV) was set as output. DL results are then integrated to MSV to revive the MC dose maps. Finally, the dose maps generated by MSV, SSV, and DL were quantitatively compared to the MC reference at both voxel level and organ level (organs at risk and lesions).

**Results:**

The DL approach showed slightly better performance (voxel relative absolute error (RAE) = 5.28 ± 1.32) compared to MSV (voxel RAE = 5.54 ± 1.4) and outperformed SSV (voxel RAE = 7.8 ± 3.02). Gamma analysis pass rates were 99.0 ± 1.2%, 98.8 ± 1.3%, and 98.7 ± 1.52% for DL, MSV, and SSV approaches, respectively. The computational time for MC was the highest (~2 days for a single-bed SPECT study) compared to MSV, SSV, and DL, whereas the DL-based approach outperformed the other approaches in terms of time efficiency (3 s for a single-bed SPECT). Organ-wise analysis showed absolute percent errors of 1.44 ± 3.05%, 1.18 ± 2.65%, and 1.15 ± 2.5% for SSV, MSV, and DL approaches, respectively, in lesion-absorbed doses.

**Conclusion:**

A hybrid transformer-based deep learning model was developed for fast and accurate dose map generation, outperforming the MIRD approaches, specifically in heterogenous regions. The model achieved accuracy close to MC gold standard and has potential for clinical implementation for use on large-scale datasets.

**Supplementary Information:**

The online version contains supplementary material available at 10.1007/s00259-024-06618-9.

## Introduction

Radiopharmaceutical therapy (RPT) has emerged as a promising approach for managing various cancers, thus enabling selective delivery of high radiation dose to the target while minimizing toxicity to normal tissues [1, 2]. Among RPT techniques, peptide receptor radionuclide therapy (PRRT) with [^177^Lu]Lu-DOTATATE has demonstrated efficacy in palliative treatment of patients diagnosed with non-resectable metastatic neuroendocrine tumors (NETs) with significant improvement in their overall survival and progression-free survival [3, 4]. The commonly used standard practice of this treatment employs “one-size-fits-all” empirical protocol, consisting of the delivery of four cycles of 7.4 GBq at 8-week intervals [5, 6]. However, this regimen may not be optimal for individual variations in tumor burden, patient physiology, body size, and overall health condition. Therefore, a personalized dosimetry and treatment planning approach is indispensable to ensure a balance between therapeutic efficacy and patient safety [7].

Implementing personalized dosimetry as a prerequisite for treatment planning in routine clinical setting poses challenges owing to (i) the lack of standardized image quantification and dosimetry protocols [8, 9], (ii) lack or weak dose-effect relationship information [10], (iii) lack of suitable radiobiological models [11], (iv) inherent low-quality and incomplete imaging data at different time points [12], (v) the heterogeneity of tumors and organs at risk (OARs), and (vi) the variability of patients’ physiology. Besides, time and labor resource requirements [11, 13] further complicate the process.

On the other hand, traditional image-based organ-level dosimetry approaches based on MIRD formalism may not suffice for patient-specific purposes as they rely on using tabulated organ-level *S*-values derived from standard phantoms [14]. These *S*-values can neither reflect activity heterogeneities within a region of interest nor do they account for inter-patient anatomical differences [15]. Voxel-level image-based dosimetry approaches have gained popularity as an alternative to overcome this limitation. Direct Monte Carlo (MC) is the consensual gold standard approach for voxel-level dose calculations which provides accurate and reliable dose estimation by considering the individual non-homogeneity of both anatomical and activity distribution into account. However, its extensive computational requirements make it impractical for routine clinical use [16–19].

To address the challenges associated with MC, various voxel-level dosimetry methods have been developed [20–22]. This includes the single *S*-value (SSV) approach using the MIRD scheme pre-tabulated *S*-values, which lacks consideration of anatomical heterogeneities as in these approaches dose calculations occurs within homogenous water medium [21]. Another approach is the multiple voxel *S*-value (MSV) technique in which instead of using a single-dose kernel calculated in soft tissue, multiple-dose kernels are used according to different tissue densities [22].

Deep learning (DL) has been successfully employed for different computational medical imaging tasks [23–29]. There have been some attempts to use DL-based voxel-wise internal dosimetry in previous studies [15, 30–33]. Lee et al. employed a U-net trained by PET and CT image patches as input to generate 3D voxel-level dose rate maps [30]. Kim et al. [33] developed a modified U-net model for voxel-wise [^177^Lu]Lu-DOTATATE dosimetry, incorporating CT and time-integrated activity (TIA) patch images as an input followed by a summation of MSV dose map for residual learning and validated their model through comparison with direct MC calculation at both organ and voxel levels. In the study by Li et al. [32], a residual deep convolutional neural network (CNN) trained with virtual patients obtained from PET images instead of SPECT/CT images was used to estimate the dose-rate maps compensated for blurring due to the poor spatial resolution of SPECT images. While the abovementioned studies benefited from utilizing CNNs, the application of transformer architectures remains limited in dosimetry tasks [34]. Transformers benefit from self-attention and can process the input data in parallel which leads to increased efficacy. In addition, they can achieve adequate performance with very limited training data [35–38].

The main objective of the present work is to evaluate a hybrid transformer-based deep learning network for voxel-level dosimetry of [^177^Lu]Lu-DOTATATE RPT in terms of computational costs and accuracy of absorbed dose calculations. To this end, we trained an MSV/DL hybrid model to predict the necessary corrections on MSV dose maps to generate MC dose maps as the ground-truth. Subsequently, DL-based absorbed dose distributions were validated by comparing the results with MC-based absorbed dose distributions. SSV and MSV dose maps were also calculated for further comparison.

## Material and methods

### Patient characteristics and data acquisition

This retrospective study included SPECT/CT images from 50 sessions of 22 patients with NETs who underwent [^177^Lu]Lu-DOTATATE therapy for up to 4 cycles. The injected activity was personalized for each cycle (median: 7363 MBq, range 1017–9657 MBq) based on kidney function, body habitus, and dosimetry results [39]. Patient characteristics are summarized in Table 1. The images were acquired on a Symbia T16 SPECT/CT camera (Siemens Healthineers, Germany) equipped with a medium-energy low-penetration collimator (MELP). The images at multiple time points were acquired at ~4 h (range 3.6–5.3), 24 h (range 19.6–25.0), 69 h (range 67.2–74.1), and 120 h post-injection. The reconstructed CT images had a matrix size of 512 × 512, acquired at 110 kVp and 126.48 ± 37.2 mAs with a voxel size of 0.9766 × 0.9766 × 5 mm^3^. SPECT images had a 128 × 128 matrix size, zoom factor of 1, and voxel size of 4.795 × 4.795 × 4.795 mm^3^. SPECT projections were obtained in 96 views (48 camera spots) with a duration of 15 s for the two first time points and 20 s for the last time points. SPECT images were reconstructed using a 3-dimensional ordered-subset expectation maximization (3D-OSEM) algorithm with 4 iterations and 8 subsets with resolution recovery, including CT-based attenuation correction, dual-energy window (DEW) scatter correction using [187.2–228.8] and [166.4–187.2] keV for photopeak and scatter windows, respectively [40]. The activity calibration, i.e., translating the reconstructed counts (count/s) into absolute activity (Bq/ml), was performed using a cylindrical phantom (Jaszczak) filled with known uniform activity concentration [41].

**Table 1:** Summary of patient demographics and treatment characteristics

Number of patients/total therapy cycles	21/55
Gender (F:M)	8 (38%):13 (62%)
Age, median (range)	62 (26–78)
Height (m), median (range)	1.69 (1.5–1.84)
Weight (kg), median (range)	76.8 (61.8–122.2)
Number of therapy cycles (1:2:3:4)	(5:5:4:7)
Injected activity (MBq), median (range)	7215 (1017–9657)

## [^177^Lu]Lu-DOTATATE dosimetry workflow

### Monte Carlo simulations

The dose maps generated through direct Monte Carlo served as standard of reference and were used as the target for training our network. For this purpose, patient-specific density maps (g/cm^3^) were derived from CT images (Hounsfield units) as described by Schneider et al. [42]. These density maps, along with time-integrated activities (TIAs) (further elaborated below), were input into a previously validated [15] MCNP MC simulator (version 2.3, Los Alamos National Laboratory) [43] generate the voxel-level absorbed dose map. About 10^8^ histories were simulated wherein the statistical uncertainty was considered negligible [44]. All calculations were performed using a 12th Gen Intel ® core^™^ i7-12700K CPU at 3.6 GHz.

### Time-integrated activities

**Fig. 1:**
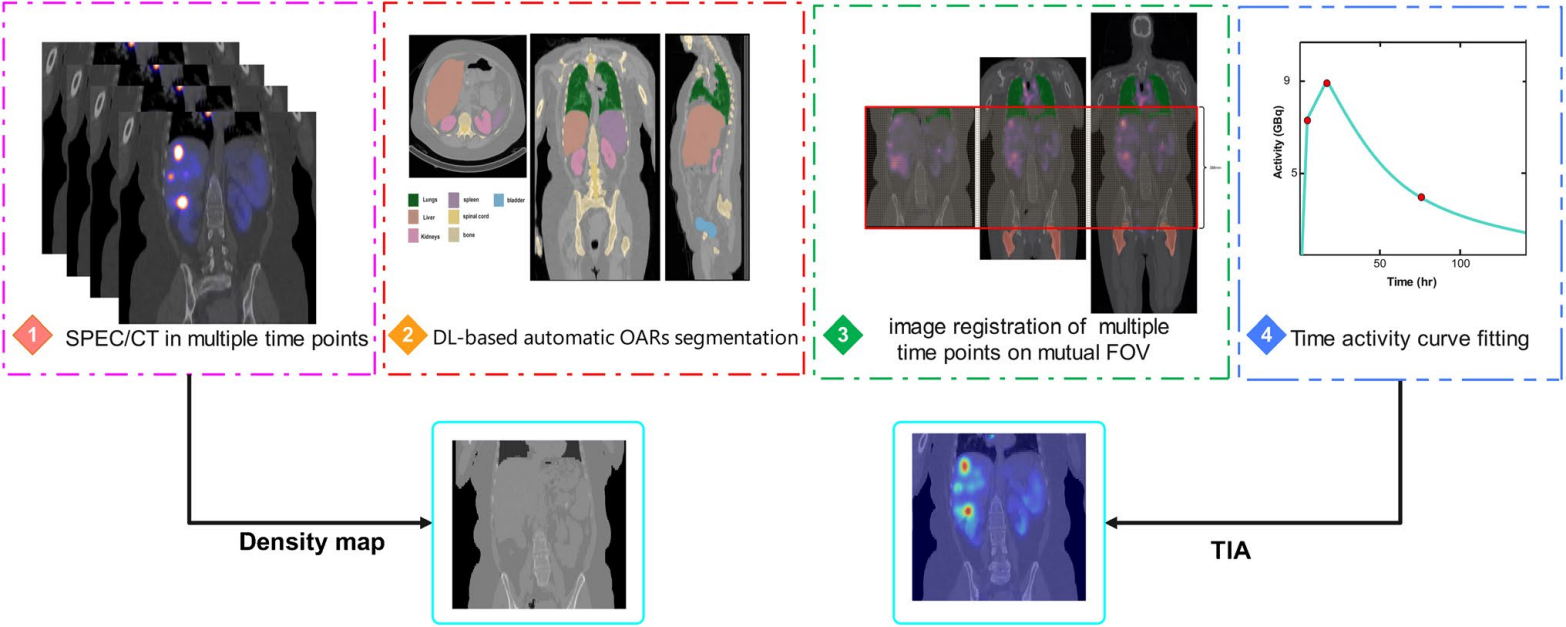
The flowchart of preparing the data for dosimetry calculations. After tumor delineation by an experienced physician and OAR segmentation using a modified UNET model, and registration of multiple-time-point SPECT images, time activity curves were calculated for each voxel, and TIA maps were extracted. These TIA maps, along with density maps, served as input for various dosimetry calculation approaches

TIAs were obtained from multiple time points of post-treatment SPECT images through the following steps:

1. A previously trained RESUNET deep learning model was used to automatically delineate organs at risk (OARs) on the CT images of hybrid SPECT/CT acquired after [^177^Lu]Lu-DOTATATE administration followed by manual adjustment of the contours. The Dice coefficients (%) for the liver, kidneys, spleen, bones, lung, and bladder were 97, 94, 95, 94, 98, and 84, respectively [45].
2. Malignant tumors were delineated on SPECT images manually by an experienced nuclear medicine specialist.
3. An intensity-based SPECT-SPECT registration with a mutual information-based cost function was applied to multiple-time-point post-treatment serial SPECT images followed by visual inspection. The registration was automated using an in-house MATLAB code based on Elastix^1^.
4. Time activity curves (TACs) were generated at the voxel level for tumors and OARs based on ^177^Lu-kinetics from multi-time-point registered SPECT images. In this regard, a trapezoid function was fitted to the TAC at each voxel for data points with time <24 h, followed by a mono-exponential function $C \left({e}^{-\lambda t}\right)$, where *C* scales the curve and *λ* is the biological clearance/elimination rate for data points (if available) with *t* > 24 h.
5. The TIA was calculated by estimating the area under the time-activity curve, using a combination of trapezoidal and exponential integrations, depending on the number of available time points, as proposed in Ref. [41]. To mitigate potential errors caused by reconstruction noise or registration issues, the effective half-lives (*T*_eff_) were determined based on the mean value of normal distribution of voxel-level $\lambda$ in the volume of interest (VOI). Figure 1 illustrates the steps taken for TIA calculation.

### Single and multiple voxel S-value (SSV and MSV) approaches

The MIRD scheme SSV and MSV dose maps were calculated for further comparisons. The voxel *S*-value kernels were generated using MCNP transport code (version 2.3, Los Alamos National Laboratory) [43]. The simulation code has been previously validated [15] and the kernels benchmarked against the database from Ref. [46]. The MSV approach involves the utilization of 8 pre-calculated voxel *S*-value kernels. These kernels are computed using the MC method within various types of tissues, including lung, adipose, soft tissue, and five different densities corresponding to bones. Subsequently, MSV dose map was calculated by the convolution of the multiple kernels into TIA according to the method proposed by Lee et al. [22].

### Network training

Before training, the CT images underwent normalization using an empirical factor of 10^3^ to reduce the dynamic range of voxel intensities and scale the Hounsfield units between 0 and 1. Similarly, the TIA images were normalized using a fixed factor of 1.5 × 10^10^ without clipping the data.

**Fig. 2:**
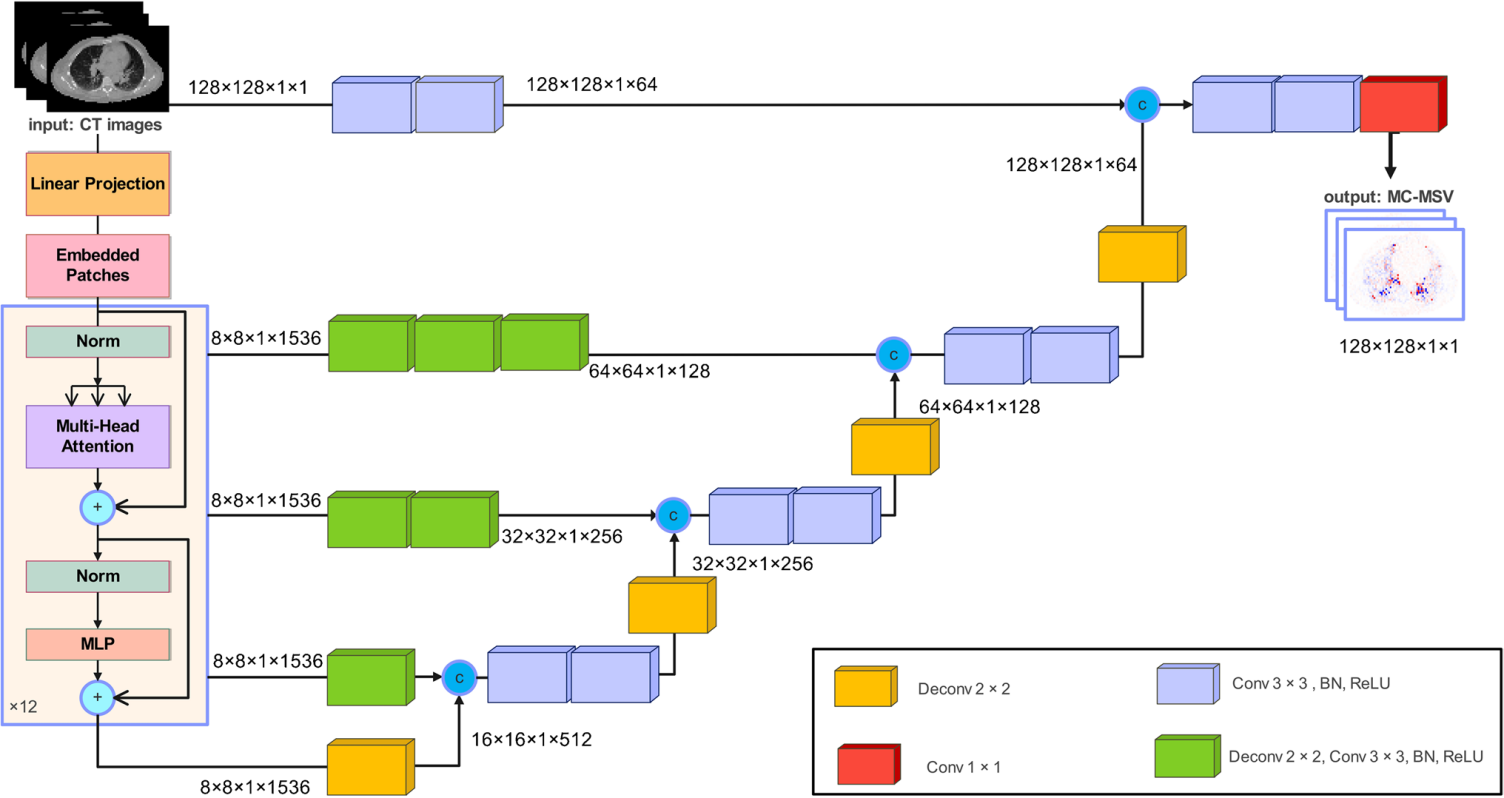
Architecture of the modified UNETR network used in this study. The architecture consists of a transformer encoder which is connected to a decoder via skip connections. The network was fed by 2D axial slices of CT images (size of 128 × 128); the output was the difference between Monte Carlo and MSV (MC-MSV)

A modified UNEt TRansformer (UNETR) architecture proposed by Hatamizadeh et al. [47] was implemented in Pytorch, and trained on an NVIDIA GeForce RTX 3080 GPU. The dataset was split into 5 sets for 5-fold cross validation ensuring each set had the same size of data, while we ensured that the multiple cycles from the same patient are never in the test and train at the same time. Training was performed using 2D axial slices. The network was fed by co-registered CT images as input with a total of 1000 epochs, initial learning rate of 0.001, and a weight decay of 0.0001. The batch size was set to 128. The mean squared error (MSE) loss function and Adam optimizer were utilized. The target of the network was the difference between MC-simulated dose maps and MSV dose maps (MC − MSV). Instead of directly generating MC dose maps, the DL output was set to be the difference between MC and MSV. Finally, MSV was added to the DL output for further evaluation (i.e., DL = MC − MSV; subsequently, MC = DL + MSV). Figure 2 displays the network architecture. We adopted this approach by hypothesizing that MSV closely approximates MC, with errors primarily limited to small heterogeneous and boundary regions. Training a model capable of correcting these errors was anticipated to contribute to performance enhancement.

We also tried multiple approaches and strategies prior to selecting the transformer-based method. First, we investigated configurations of modified UNet by using versions of MONAI DynUNet class [48, 49]. Following unsatisfactory performance, we employed U2-Net architecture [50] by using two inputs of CT + TIA to skip the need for MSV calculations in MC dose map prediction. As the results were not conclusive, another strategy was adopted in which CT + MSV was alternatively used as inputs to predict the MC dose map. The initial settings (learning rate, decay, number of epochs, etc.) were the same as those utilized in the current model. Moreover, we attempted multiple configurations of 2D axial slices and 3D boxes as inputs to the model. However, these strategies did not yield the expected outcomes.

### Evaluation strategy

The DL-generated and calculated dose maps using the two MIRD formalisms, namely, SSV and MSV, were quantitatively compared to the reference MC dose maps using various metrics. These include voxel-wise root mean square error (RMSE), mean square error (MSE), mean absolute error (MAE), mean error (ME), structural similarity index (SSIM), peak signal-to-noise ratio (PSNR), relative error (RE), and relative absolute error (RAE). Additionally, Gamma index evaluation was performed with the criteria of 4.795-mm distance to agreement (DTA) and 1% dose difference (DD). The Gamma pass rates and Gamma maps were calculated to assess the dose distribution agreement between DL, MSV, and SSV and the MC reference. The pass rates were calculated according to Eq. (1):

1 $$Pass\;rate\;\left(\%\right)\;=\frac{Number\;of\;voxels\;wih\gamma\leq1}{Total\;number\;of\;voxels}\;\times\;100$$

Moreover, organ-wise absorbed doses were compared among all the mentioned strategies in terms of organ error (%) and organ absolute error (%). For qualitative evaluation, dose volume histograms (DVHs) were compared with the reference, i.e., Monte Carlo.

## Results

**Fig. 3:**
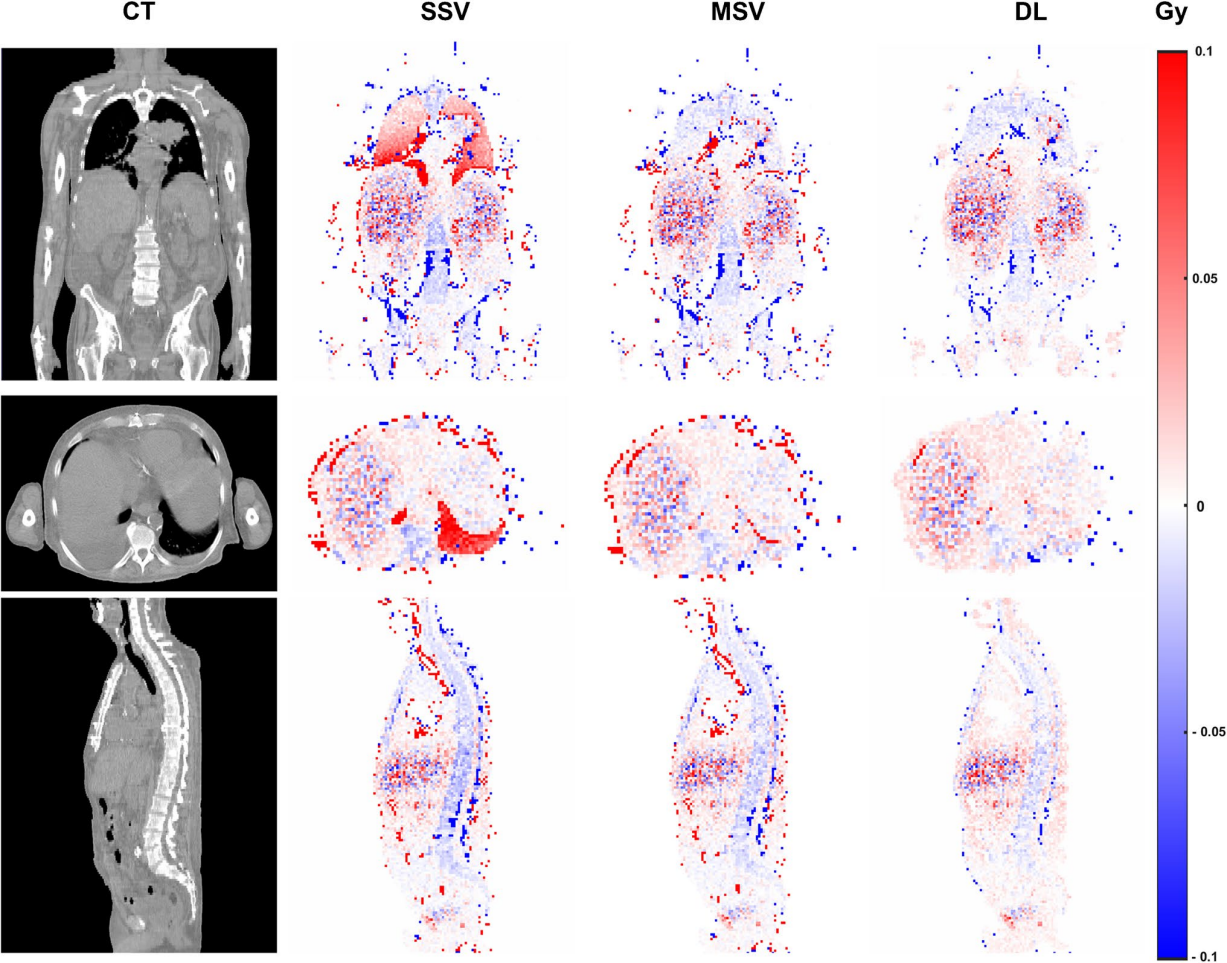
Representative relative absolute error maps in coronal, axial, and sagittal views. For each dosimetric approach, the errors were calculated with respect to Monte Carlo simulations serving as standard of reference. The SSV method tends to overestimate absorbed dose values within low-density regions (e.g., lungs), while overestimating this quantity in high-density structures (bones). The errors were reduced when utilizing the MSV method. However, our DL approach demonstrated more effective error mitigation

**Fig. 4:**
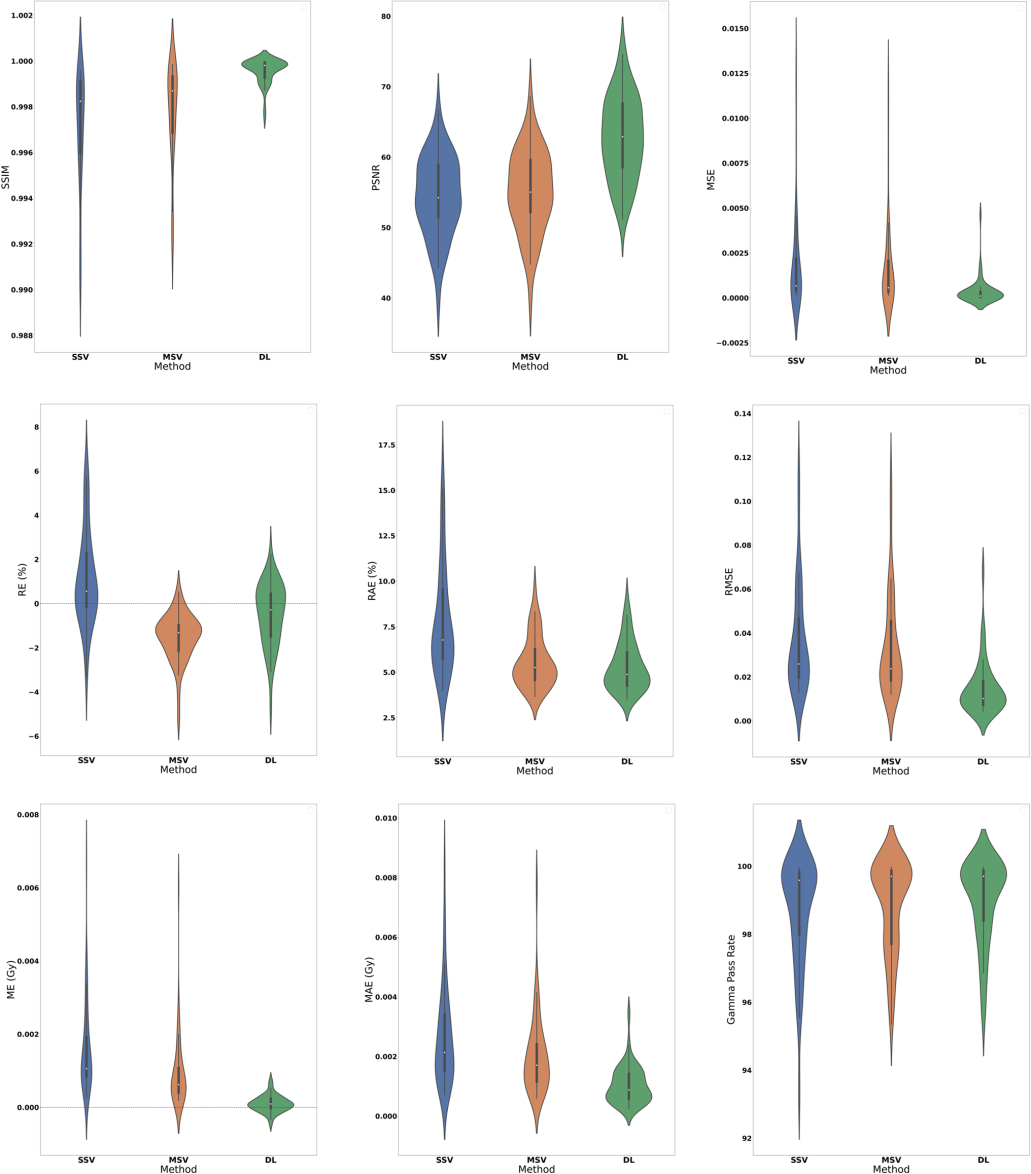
The voxel-wise quantitative metrics distributions calculated for three dosimetric approaches with respect to MC. Our deep learning (DL) model exhibited superior performance, as evidenced by high-value metrics like SSIM, PSNR, and Gamma pass rates. Additionally, the model effectively reduced errors, as indicated by metrics, such as MSE, ME, MAE, and RMSE

Only tumors with volumes >2 ml (mean volume: 38.18 ± 79.4 ml, range: 2.2–947.2 ml) were delineated in this study to be included in the analysis. The mean absorbed dose values (Gy) of tumors and OARs for each patient, calculated from MC simulations for the first treatment cycle, are presented in Supplementary Table 1. Figure 3 displays the voxel-wise relative absolute error maps, highlighting the majority of errors in lung tissue when using the SSV dose calculation approach. Voxel-wise quantitative metrics for three dosimetry approaches with respect to MC are tabulated in Table 2. Our DL approach achieved the lowest errors (e.g., RMSE, ME, MAE, MSE, RE, RAE) and maximum resemblance representatives (e.g., SSIM, PSNR, Gamma pass rates). The distribution of these quantitative metrics is visualized using violin plots in Fig. 4. Additionally, as a result of employing the 5-fold cross-validation approach, these quantitative metrics and their distribution are reported separately for each fold in Supplementary Table 2 and Supplementary Fig. 1, respectively.

**Table 2:** The voxel-wise quantitative metrics calculated for 3 dosimetric approaches with respect to MC calculations

	SSIM (%)	PSNR	RMSE (Gy^2^)	ME (Gy)	MAE (Gy)	MSE (Gy^2^)	RE (%)	RAE (%)	Gamma pass rates (%)
DL	99.96 ± 0.0005	62.70 ± 5.79	0.015 ± 0.01	0.0001 ± 0.0002	0.0010 ± 0.0006	0.0004 ± 0.0007	-0.46 ± 1.38	5.28 ± 1.32	99.0 ± 1.20
MSV	99.78 ± 0.0022	55.13 ± 5.78	0.034 ± 0.02	0.0009 ± 0.0010	0.0020 ± 0.0013	0.0017 ± 0.0025	-1.50 ± 1.05	5.54 ± 1.40	98.80 ± 1.30
SSV	99.71 ± 0.0025	54.42 ± 5.6	0.036 ± 0.02	0.0016 ± 0.0012	0.0026 ± 0.0016	0.0019 ± 0.0027	1.13 ± 2.15	7.8 ± 3.02	98.71 ± 1.52

The values are reported as average ± their corresponding standard deviations. *SSIM*: structural similarity index, *PSNR*: peak signal-to-noise ratio, *RMSE*: root mean squared error, *ME*: mean error, *MAE*: mean absolute error, *MSE*: mean squared error, *RE*: relative error, *RAE*: relative absolute error, *DL*: deep learning, *MSV*: multiple *S*-value, *SSV*: single *S*-value

**Fig. 5:**
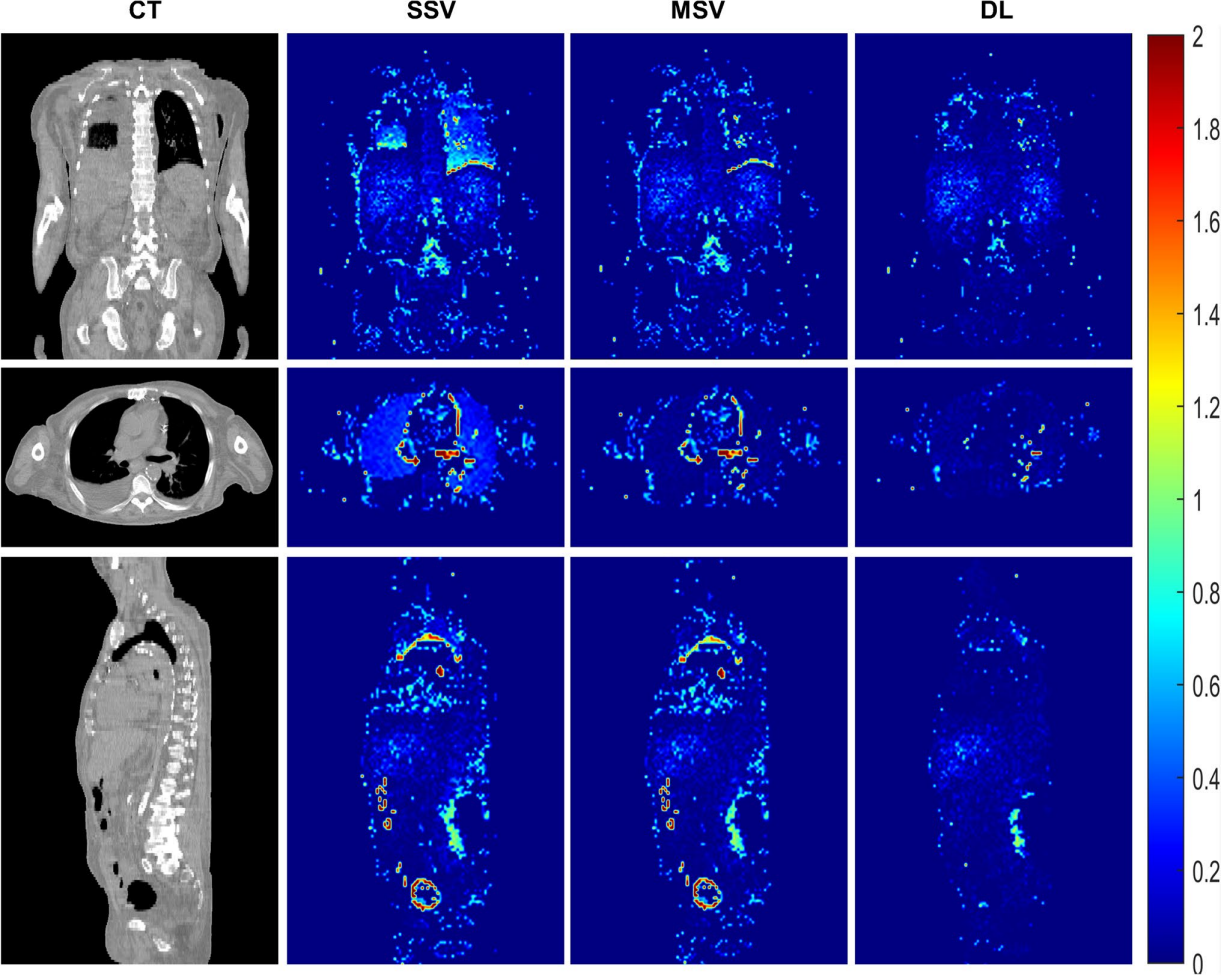
Gamma maps in coronal, axial, and sagittal view for a case with pass rates of 98% for SSV 99% for MSV and 99.5% for DL approaches, with the criteria of 4.79 mm DTA and 1% DD. Voxel values less than or equal to 1 passed for this evaluation

We conducted a 3D Gamma evaluation considering MC dose maps as standard of reference. Figure 5 presents an example of Gamma maps with the highest number of rejected voxels (Gamma value >1) in the SSV approach. Our hybrid DL approach, on the other hand, generated dose maps with reduced number of rejected points in this evaluation. Furthermore, we performed the Gamma evaluation within the lesion volumes, and the pass rates were found to be 96.8 ± 6%, 97.2 ± 5.3%, and 97.35 ± 5.1% for SSV, MSV, and hybrid DL approaches, respectively.

**Fig. 6:**
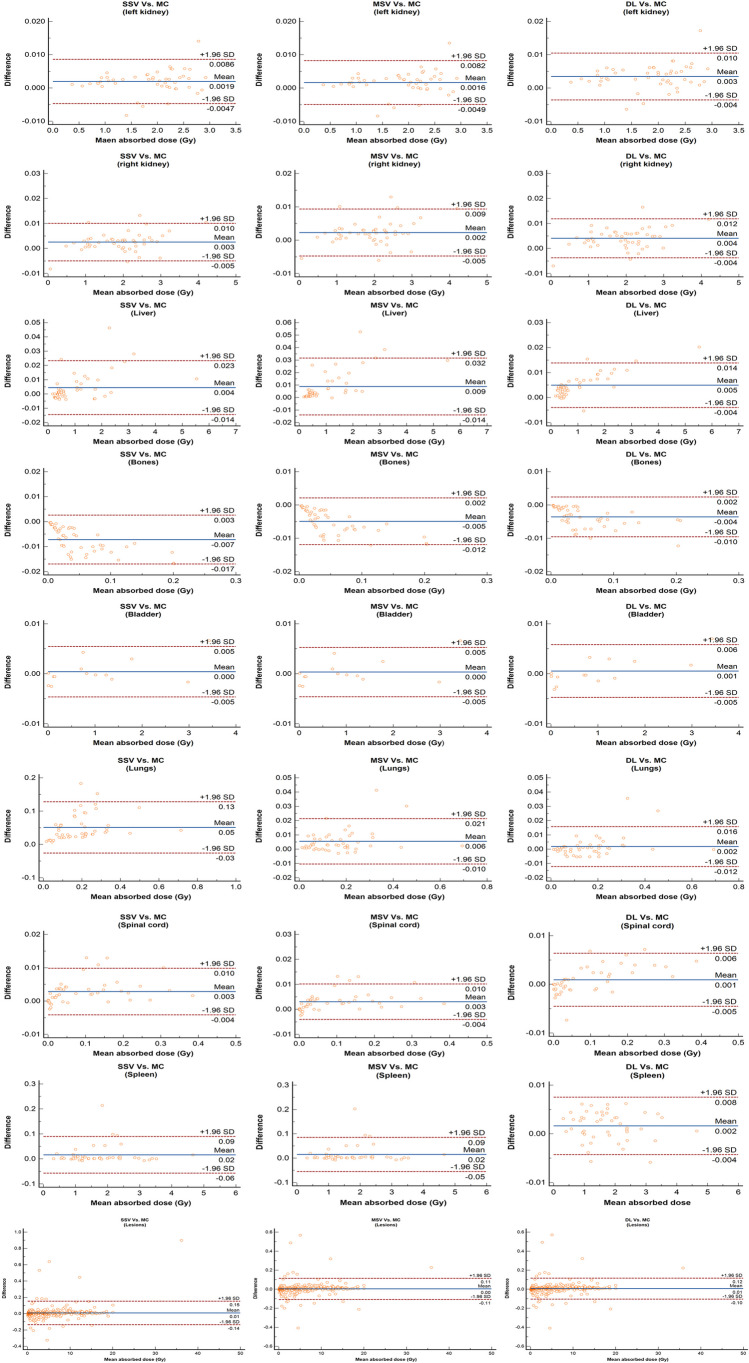
Bland-Altman plots demonstrating the differences of each dosimetric approach with respect to MC for OARs and lesions. The solid line represents the mean difference with MC, whereas the dashed lines indicate the upper and lower limits of agreement (mean difference ± 1.96 times the standard deviation of the differences

Additionally, we reported the organ-level errors for lesions and OARs in Table 3. Our hybrid DL approach showed a reduction in error values for most organs, except for the left kidney. Organ-wise errors for each fold are also reported in Supplementary Table 3. The Bland-Altman plots in Fig. 6 depict the differences in mean absorbed dose calculated by each dosimetry approach with respect to MC for OARs and lesions. The solid line represents the mean difference with MC, whereas the dashed lines indicate the upper and lower limits of agreement (mean difference ± 1.96 times the standard deviation of the differences). An example of the DVH for lesions and various OARs is depicted in Supplementary Figure 2.

**Table 3:** Organ-wise errors for each dosimetric approach with respect to MC for lesions and OARs

	Error (%)			Absolute percent error (%)		
	SSV	MSV	DL	SSV	MSV	DL
Lesions	0.06 ± 3.38	−0.13 ± 2.90	0.05 ± 2.70	1.44 ± 3.05	1.18 ± 2.65	1.15 ± 2.5
Left kidney	0.11 ± 0.12	0.09 ± 0.12	0.29 ± 0.15	0.16 ± 0.05	0.14 ± 0.05	0.30 ± 0.10
Right kidney	−0.12 ± 1.95	−0.04 ± 1.31	0.01 ± 1.67	0.45 ± 1.9	0.35 ± 1.26	0.49 ± 1.59
Liver	0.90 ± 0.94	0.83 ± 0.86	0.59 ± 0.42	0.90 ± 0.93	0.84 ± 0.86	0.61± 0.40
Spleen	1.05 ± 2.18	0.99 ± 2.08	0.14 ± 0.26	1.11 ± 2.1	1.06 ± 2.05	0.22 ± 0.20
Bones	−17.07 ± 9.32	−11.47 ± 6.4	−8.21 ± 5.92	17.07 ± 9.32	11.47 ± 6.4	8.21± 5.92
Lungs	45.0 ± 39.1	4.57 ± 5.86	0.49 ± 3.52	45 ± 39.1	4.84 ± 5.64	2.60 ±2.40
Spinal cord	3.25 ± 14.98	3.70 ± 14.65	−4.50 ±15.44	10.1 ± 11.44	9.96 ± 11.27	7.42 ±14.25
Bladder	−2.0 ± 7.19	−2.03 ± 7.17	−0.79 ± 1.97	2.44 ± 7.03	2.42 ± 7.04	1.08 ±1.81

The average values are reported with ± SD

### Computational time

The computational time of the different strategies was compared by computing a dose map corresponding to the 512 × 512 × 130 SPECT/CT image size on a desktop with 32 GB of RAM, CPU (12th Gen Intel ® core^™^ i7-12700K at 3.6 GHz) and GPU (NVIDIA GeForce RTX 3080). Since the calculation of TIA and automatic organ segmentation was common among all strategies, the corresponding time for these steps was not taken into account. For the DL dose map generation, including pre-processing (CPU), inference (GPU), and post-processing (CPU), the process took approximately 1 min. While for the whole inference on CPU for desktops not equipped with dedicated GPU, the whole computational time was 4 min. On the other hand, the dose map calculation based on SSV and MSV took ~1.2 h each on CPU. However, MC calculation was the most time-consuming technique, taking about 2 days on CPU to simulate 100 million histories. 

## Discussion

Fast and accurate generation of dose maps may help the implementation and adoption of personalized dosimetry in RPT in the clinic. A modified UNETR model combined with MSV was developed to achieve efficient and accurate voxel-wise dosimetry for [^177^Lu]Lu-DOTATATE therapy. This study evaluated organ- and voxel-wise errors in comparison to MC dose calculation, considered as standard of reference. While this study does not aim to conduct a direct head-to-head comparison of independent approaches, we included the analysis of the voxel *S*-value MIRD formalisms to ensure a comprehensive dosimetry investigation. Additionally, our objective is to highlight and demonstrate the potential limitations associated with these approaches. One of the motivations behind employing a DL network for [^177^Lu]Lu-DOTATATE voxel-wise dosimetry was the limitation of current voxel-level MIRD formalisms. In the SSV approach, the dose map from a radiopharmaceutical is constructed through the convolution of only one voxel *S*-value kernel (water) with the TIA calculated from emission tomography images (PET or SPECT). This approach does not account for organ heterogeneities [21]. The alternative MIRD-based dosimetry approach, referred to as multiple *S*-value (MSV), utilizes multiple kernels, overcoming the limitations of the SSV technique. However, it still presents visible errors in tissue boundaries and interfaces [22].

Monte Carlo simulations were used as the gold standard. However, MC itself is prone to statistical errors and differences with real-world occurrences that may lead to missing dose heterogeneities. We had access to limited computational resources, and hence, only 100 million tracked particles were simulated which means the MC itself is prone to sampling statistical errors even negligible [44]. Besides, the inherent approximations within MC calculations and geometry simplifications may not accurately capture the complexity of clinical scenarios.

In a broader point of view, the distinctions among SSV, MSV, and MC techniques lie in the extent of approximations and assumptions made. SSV assumes uniform radiation absorption across all tissues (assumed as water), whereas MSV introduces tissue-specific kernels, reducing assumptions but increasing the descriptive parameters and calculations. In contrast, MC makes the fewest assumptions but requires more parameters to be defined. This explains the superior performance of MSV over SSV and even better performance of MC over MSV.

Noteworthy, absorbed dose heterogeneity in the context of RPT includes both spatial and temporal heterogeneities. The first refers to uneven distribution of absorbed doses within a VOI, while the second refers to the variation of absorbed dose rates over time due to pharmacokinetics and radioactive decay. Although dynamic imaging or imaging in multiple time points is performed to address the temporal heterogeneity, radiopharmaceutical kinetics is oversimplified in TAC fitting process [51]. The accuracy of TAC derivation depends on the spatial resolution of the imaging modality as well as the frequency of the time points. Low spatial resolution or infrequent time points lead to inadequate capturing of rapid changes in radiotracer distributions, affecting the accuracy of TACs and, consequently, the calculated absorbed doses. However, the initial steps including time-point registration, curve fitting, and TIA calculations were similar for all four dose calculation methods (SSV, MSV, MC, and DL) evaluated in this work.

Most of the errors in voxel-wise dose calculations occur when using the SSV approach, which is evident in Fig. 3. The SSV method overestimates the absorbed dose values inside the low-density tissues, such as the lungs, and underestimates the absorbed dose values in tissues with high density, such as bones. While MSV mitigated these errors, it still showed limitations in high-gradient tissue density regions, i.e., the tissue boundaries (lung/soft tissue and bone/soft tissue). However, our proposed hybrid DL model effectively reduced errors in these areas as confirmed by Gamma maps (Fig. 5). Our method improves the MSV dose map accuracy both in voxel-level and organ-level evaluations as presented in Figs. 4 and 6. Although Gamma evaluation pass rates were reported, displaying Gamma maps allowed observing regions of failure. In addition, lesion-wise Gamma evaluation indicated a slightly higher pass rate for the DL approach. To the best of our knowledge, there is no guideline indicating a specific criterion for Gamma evaluation in targeted radiotherapy. However, the chosen criteria (DTA = 4.79 mm and DD = 1%) were based on the spatial resolution of SPECT images as a determinative factor and external beam radiation therapy standards.

DVHs are shown in supplementary Figure 2, where we can compare the dose distributions visually. The rationale behind using DVHs relies on their ability to provide a comprehensive and quantitative summary of dose distribution. This involves representing the percentage volume of a volume of interest (VOI) that receives a specific dose or higher (*V*_*x*_ (%)) or a specific absorbed dose received by a percentage of volume (*D*_*x*_ (Gy)). This quantitative data is crucial to evaluate the delivered absorbed doses to lesions and OARs, especially for heterogenous dose distributions. DVHs visually depict dose distribution, facilitating straightforward comparisons between different dose distributions. Although DVHs are well-studied for handling heterogeneous dose distributions, they are not able to provide spatial information on where dose inhomogeneities occur within the volume. Furthermore, by simplifying the complex 3D dose distribution into a single metric for each dose level, DVHs may overlook specific geometric patterns or gradients that hold clinical significance. Additionally, the granularity of a DVH depends on the selected dose-volume bin size, and slight changes in bin size can alter the shape of the DVH curve, potentially influencing interpretations [52]. One of the reasons that prevented us from distinguishing the differences among various dosimetry approaches using DVHs is attributed to the absence of spatial information. To address this limitation, we have employed Gamma analysis and visualized the results to identify specific regions showing observed differences.

In this study, a transformer-based UNET-shaped model was utilized. To our knowledge, in dosimetry-related studies, CNNs have been mostly utilized, while the application of transformers in this specific task remains limited [34, 5 4]. Transformer-based models are generally used in Natural Language Processing (NLP) with the capability of highlighting the important features of word sequences due to self-attention mechanism [47, 54]. The computational efficiency and superiority of our model in image domain-related tasks, such as organ segmentation, has been demonstrated compared to state-of-the-art CNNs [34, 47]. While the optimal performance of complex transformers often relies on the availability of a large-scale dataset, our model as a less-complex transformer model does not necessarily perform better using larger-scale dataset. It should be mentioned that the true value of a large-scale dataset lies in its capacity to inject diversity into the training process. The absence of diversity in a large-scale dataset may result in suboptimal performance when applied on external datasets. In our study, despite the limited size of our dataset, we paid attention to diversity by including various patients with personalized injected activities ranging from 1017 to 9657 MBq. While transformer-based models may not consistently outperform other networks in terms of performance metrics, their adaptability to small-size datasets, proficiency in managing diversity, and ability to capture complicated relationships make them well-suited for personalized dosimetry tasks. Nevertheless, to cope with the inherent black-box nature of deep learning methods, including transformers, the development of explainable AI models to enhance the interpretability and trustworthiness of the predicted results is suggested [55].

It is worth mentioning that before opting for the current approach, we tried other strategies involving the use of other networks, such as UNET and U2-NET. However, the results were not as favorable as using the adopted network. The best results we could achieve through other networks or strategies are summarized in Supplementary Table 4.

Noteworthy, the size, shape, and texture variations of organs/lesions, as well as SPECT and CT images, in different regions of interest, can influence the performance of the DL method, justifying distinct errors in various regions of interest.

Despite using limited training data, our hybrid model provided fast and accurate voxel-wise dose estimations. Dose map generation time for an image size of 128 × 128 × 80 voxels is approximately 1 min, which is an impressive improvement compared to MC running time. The model performance was consistent among folds showing robustness. Moreover, the DL approach exhibited a slight advantage over MIRD-based dose calculations, consistent with recent studies on [^177^Lu]Lu-DOTATATE dosimetry calculated by DL [33, 53]. In this study, rather than directly generating MC dose maps, we calculated the difference between MC and MSV which resulted in further error reduction. Since MSV provides a good approximation of MC dose maps, and errors only occur in small heterogeneous and boundary regions, therefore, training a model that focuses on correcting these errors helped in performance improvement. The network predicts the difference map based on anatomical information in the CT image. Although generating the difference dose map between MC and MSV requires the MSV dose map calculations to perform the DL-based dosimetry calculation, once the model has been trained, the inference time (~1 min including pre- and post-processing) is negligible compared to the MSV calculation time (~1.2 h). Nevertheless, it is important to highlight the dependence of the proposed approach on the MSV map post-inferencing to reconstruct the MC dose map, as outlined in the “Material and methods” section (predicted MC = DL + MSV). Therefore, the DL model is presented as a hybrid MSV/DL model, and the inference time should be added to MSV calculation time (1.2 h on CPU + 1 min). As described in the “Material and methods” section, the MSV calculations were performed on CPU. We implemented the MSV calculation part on GPU to accelerate the procedure. Indeed, GPU-based computations for MSV would further reduce the calculation time (14 s for each voxel *S*-value kernel of MSV for a 2-bed SPECT image with a resolution of ~4.8 mm traceable on P#6 of SNMMI dosimetry challenge dataset [13]). Therefore, conducting our hybrid DL approach on GPU would take ~3 min for a two-bed SPECT (MSV with 8 kernels ~2 min, and inference time ~1 min).

Automated tools, such as OAR segmentation, which is the most time-consuming step in the dosimetry workflow, and SPECT-SPECT registration facilitated and accelerated our study. The OAR segmentation model demonstrated a high degree of reliability as reflected by the performance metrics (high Dice coefficients) [45]. This model can be employed in dosimetry workflow of other radionuclides and embedded in treatment planning systems. It should be mentioned that organ segmentation on CT images is a more practical approach, yet susceptible to potential errors arising from the different resolutions of CT and SPECT images, the spill-in/out phenomena in SPECT images, and the likely mismatch between SPECT and CT images. Lesion segmentation was manually carried out by a physician, considering both SPECT and CT images, along with the fused image at all three time points.

The SPECT-SPECT registration for the generation of TIA has an advantage over transformation methods based on the CT of hybrid SPECT/CT, avoiding issues with mis-alignment errors in SPECT-CT co-registered images originating from patient movement and respiratory motion [44, 56]. However, given the time intervals between different acquisitions, several factors introduce challenges to achieving precise alignment even with SPECT-SPECT registration approach. These factors include patient motion, fluctuations in radiopharmaceutical distribution, radioactive decay, inherent image noise and poor spatial resolution, lack of anatomic information, and anatomical-related variables, such as nutritional and voiding status of the patient. The complicated interplay of these dynamic elements necessitates a comprehensive appraisal to ensure accurate and reliable image registration [56]. The incorporation of trapezoidal and mono-exponential TAC fitting in this study allowed modeling both the uptake and washout of the tracer, which is not achievable by using mono-exponential TAC fitting alone [13], particularly in organs at risk, such as the kidneys [41].

This study bears inherently a number of limitations, including the small sample size. Yet, the results demonstrated the effectiveness of the network with a 5-fold cross-validation strategy. Furthermore, the unavailability of diagnostic images for accurate tumor delineation may lead to underestimation of absorbed dose values [13]. Manual lesion segmentation on SPECT/CT images introduces inherent sources of error, particularly in cases where the lesions are distributed across various anatomical regions. The reliance on visually selected image windowing (window width and window level) poses challenges in accurately defining lesion borders. Such misalignments adversely impact the accuracy of TAC calculation, as the delineated lesion may include voxels from surrounding tissues. Conversely, there is a possibility of missing part of the lesion when using this approach.

Another limitation of our study is that users are required to compute the MSV dose maps for reconstructing MC estimate from DL results. Nevertheless, the MSV dose map can serve as a conventional method with a known extent of error, aiding in identifying cases where the DL dose map prediction may be unreliable in an external dataset. The time required for MSV calculation contributes to the safety of our methodology in identifying outliers in an external, unseen dataset. Another limitation of this work is the use of mono-centric instead of multi-institutional datasets to ensure the robustness and generalizability of trained hybrid DL model, and as such, further evaluation on larger multi-centric datasets is still required.

## Conclusion

This work aimed at developing a hybrid transformer-based deep learning model incorporating the MSV approach, trained for fast and accurate generation of absorbed dose maps. The results demonstrated superiority of this hybrid network over the MIRD SSV approach, and a slight improvement compared to the MSV approach while significantly outperforming MC in terms of computational efficiency. It can be concluded that our method achieved an accuracy close to the gold standard and surpassed it in terms of time and computational effort, making it more readily applicable in clinical setting once trained using a large-scale and multi-centric dataset. In summary, according to our results, using the MSV method is recommended for organ-level dosimetry, whereas MC or hybrid DL method is suggested for voxel-wise dosimetry, especially for small lesions/organs in regions with high gradient of density.

### Supplementary Information

Below is the link to the electronic supplementary material.

Supplementary file1 (PDF 621 KB)

## Footnotes

1 https://elastix.lumc.nl/
