# Supplementary material for: Deep transformer-based personalized dosimetry from SPECT/CT images: a hybrid approach for [177Lu]Lu-DOTATATE radiopharmaceutical therapy
Source: Eur J Nucl Med Mol Imaging. 2024 Jan 25;51(6):1516–29. doi: 10.1007/s00259-024-06618-9 (PMC11043201; doi:10.1007/s00259-024-06618-9)
Supplement: Supplementary file 1 — Supplementary file1 (PDF 621 KB) [file 259_2024_6618_MOESM1_ESM.pdf]

Supplementary Table 1. The administered activities are calculated in a personalized way and mentioned only for cycle one. Mean absorbed doses are calculated using Monte Carlo (Gy) for the corresponding first cycle. Not all tumors are mentioned. The volumes (ml) of tumors are reported in parentheses. The tumors are located in the liver or other soft-tissues except * which are on bony structures and ** which is in lung tissue. The dose information is not provided for those whose scan FOV does not cover the bladder. Patients N-026 and N-028 had only one tumor.

Patient code	Sex	Age(y)	Height (m)	Weight (Kg)	Number of therapy cycles	Admin. Activity (Mbq)	Left kidney	Right kidney	Healthy liver	Spleen	Bone marrow	Bladder	Lungs	Spinal cord	Tumor 1	Tumor 2
N-026 (20161108)	F	26	1.64	122.18	4	1017	1.645	3.187	0.49	2.69	0.026	_	0.00032	0.035	2.2 (7)	-
N-014 (20160712)	F	40	1.7	61.82	4	9213	0.837	0.904	0.33	0.95	0.136	0.377	0.001	0.286	0.7 (52.6)	1.07 (30.9)
N-027 ((20161213)	F	55	1.6	81.8	2	6105	0.803	1.22	1.586	0.761	0.086	0.0036	0.0001	0.105	7 (16.7)	9.16 (354)
R-011 (20160607)	M	57	1.8	90.9	1	1206	1.1	0.921	3.01	0.975	0.921	_	0.0002	0.004	6.7 (60)	10 (947)
N-015 (20160712)	M	58	1.64	68.64	4	1102	1.618	0.792	2.37	0.9464	0.011	_	0.147	0.064	1.26 (60)	4.75 (17.6)
N-018 (20160816)	M	58	1.65	77.73	3	9324	1.99	2.05	1.71	3.37	0.029	_	**8.84**	0.005	10.65 (15.3)	8.19 (81.6)
N-010 (20160607)	M	60	1.8	80.45	4	9250	2.27	2.424	0.47	1.0404	0.098	_	0.118	0.14	2.5 (48)	0.34 (64.6)
N-029 (20170131)	M	62	1.7	93.5	1	5846	0.7	0.77	1.127	4.32	0.0197	0.0521	0.0007	0.001	20 (83.6)	11.3 (18.9)
N-020 (20160823)	M	62	1.67	61.36	1	8658	0.928	1.07	**5.57**	1.006	0.1217	_	0.1366	0.1217	15.92 (7.4)	10.3 (200.7)
R-009 (20160607)	M	64	1.71	120.9	2	1043	1.902	1.404	0.96	2.71	0.013	_	0.0001	0.0031	9 (384)	11 (60)
N-033 (20170221)	F	68	1.69	63.6	1	7992	1.997	1.896	1.03	2.265	0.201	0.60	0.025	0.237	7.7* (9.1)	12.8 (43.5)
R-021 (2010823)	M	71	1.65	88.07	2	9657	2.18	2.335	0.431	1.996	0.08	_	0.049	0.08	2.7 (33.6)	0.56 (3.8)
N-025 (20161108)	M	74	1.7	74.9	2	7215	2.03	1.74	2.04	1.856	0.11	**2.117**	0.02	0.189	3.1** (56.8)	0.68 (3.5)
N-030 (20170214)	F	75	1.56	63.2	1	7881	2.207	2.66	0.65	2.25	0.084	0.437	0.007	0.162	7.4 (29.3)	6.95 (27.3)
N-019 (20160823)	M	78	1.83	75.9	2	5698	0.728	0.688	0.353	0.842	0.0379	_	0.0028	0.089	0.25* (4.6)	1.2* (3.3)
N-023 (20160927)	M	Na	1.69	85.9	4	9612.53	1.618	1.5708	2.37	0.9464	0.069	_	0.147	0.064	14.9 (92)	12 (120)
N-028 (20170117)	F	Na	1.62	72.3	3	9401.65	2.275	2.6	0.574	1.4	0.004	_	0.015	0.098	12.6 (100.7)	-
N-038 (20170718)	F	Na	1.5	76.8	3	8258	1.85	1.635	0.358	0.856	0.117	0.512	0.001	0.242	5.2* (48)	1.13* (6)
N-044 (20170613)	M	Na	1.78	79.9	4	4900.2	0.30	0.398	0.17	0.374	0.0038	_	0.0006	0.00051	1.44 (120)	0.8 (37)
N-045 (20170613)	F	Na	1.65	68.6	4	4664.17	0.732	0.916	0.274	0.527	0.0247	_	0	0.018	1.06 (44)	11.3 (107)
N-046 (20170613)	M	Na	1.84	71.2	3	10261	0.86	1.087	0.277	0.556	0.555	_	0.089	0.079	14.3 (265)	8.85 (76)

Supplementary Table 2. The voxel level quantitative metrics for SSV, MSV and DL approaches in each fold. The reported values represent the mean ± SD.

	Fold 1			Fold 2			Fold 3			Fold 4			Fold 5		
	**SSV**	**MSV**	**DL**	**SSV**	**MSV**	**DL**	**SSV**	**MSV**	**DL**	**SSV**	**MSV**	**DL**	**SSV**	**MSV**	**DL**
SSIM (%)	99.78 ± 0.0012	99.83 ± 0.0011	99.97 ± 0.0002	99.70 ± 0.0029	99.75 ± 0.0026	99.97 ± 0.0004	99.73 ± 0.0021	99.80 ± 0.0018	99.97 ± 0.0004	99.61 ± 0.0031	99.70 ± 0.0026	99.94 ± 0.0007	99.73 ± 0.0029	99.81 ± 0.0024	99.95 ± 0.0007
PSNR	54.3502 ± 4.5518	55.02 ± 4.80	61.82 ± 4.84	54.90 ± 4.34	55.49 ± 4.38	65.41 ± 3.95	54.50 ± 4.81	55.26 ± 4.8	62.76 ± 6.13	52.36 ± 7.37	53.03 ± 7.6	61.97 ± 6.82	55.95 ± 6.7	56.85 ± 7.05	61.47± 6.84
RMSE (Gy^2)	0.03 ± 0.01	0.02 ± 0.01	0.01 ± 0.01	0.04 ± 0.03	0.04 ± 0.03	0.01 ± 0.0075	0.03 ± 0.02	0.03 ± 0.02	0.01 ± 0.01	0.03 ± 0.01	0.03 ± 0.01	0.01 ± 0.0053	0.03 ± 0.02	0.02 ± 0.02	0.01 ± 0.01
ME (Gy)	0.0013 ± 0.0011	0.0007 ± 0.0005	0.0001 ± 0.0001	0.0021 ± 0.0018	0.0015 ± 0.0016	0.0002 ± 0.0001	0.0018 ± 0.0013	0.0011 ± 0.0010	0.0000 ± 0.0003	0.0014 ± 0.0008	0.0008 ± 0.0006	-0.0001 ± 0.0002	0.0014 ± 0.0009	0.0006 ± 0.0006	0.0002 ± 0.0002
MAE (Gy)	0.0022 ± 0.0016	0.0016 ± 0.0010	0.0009 ± 0.0006	0.0032 ± 0.0022	0.0026 ± 0.0020	0.0010 ± 0.0005	0.0029 ± 0.0019	0.0022 ± 0.0014	0.0011 ± 0.0009	0.0024 ± 0.0012	0.0018 ± 0.0008	0.0009 ± 0.0005	0.0025 ± 0.0012	0.0018 ± 0.0008	0.0012 ± 0.0005
MSE (Gy^2)	0.0012 ± 0.0014	0.0010 ± 0.0012	0.0003 ± 0.0006	0.0033 ± 0.0043	0.0030 ± 0.0039	0.0002 ± 0.0002	0.0019 ± 0.0017	0.0016 ± 0.0015	0.0004 ± 0.0005	0.0014 ± 0.0015	0.0013 ± 0.0014	0.0001 ± 0.0001	0.0016 ± 0.0034	0.0014 ± 0.0031	0.0007 ± 0.0014
RE (%)	1.22 ± 1.70	-1.23 ± 0.56	0.32 ± 0.55	1.01 ± 2.28	-1.63 ± 0.66	0.57 ± 0.60	1.43 ± 2.24	-0.9 ± 0.9	-1.48 ± 0.9	0.72 ± 2.37	-2 ± 1.41	-1.81 ± 1.4	1.27 ± 2.42	-1.7 ± 1.23	0.13 ± 1.21
RAE (%)	7.05 ± 3.61	5.09± 1.73	4.67 ± 1.65	8.31 ± 3.34	5.84 ± 1.37	5.19 ± 1.28	7.60± 3.11	5.21 ± 1.30	5.40 ± 1.27	7.72 ± 3.3	5.7 ± 1.67	5.64 ± 1.56	8.21 ± 2.12	5.8 ± 0.9	5.44 ± 0.78
Gamma pass rate (%)	98.86 ± 0.94	98.87± 1.01	99.19 ± 0.66	99.19 ± 1.25	99.22 ± 1.24	99.5 ± 0.67	98.38 ± 2.15	98.98 ± 1.5	98.93 ± 1.51	98.22 ± 1.68	98.3 ± 1.50	98.30 ± 1.47	98.9 ± 1.37	98.93 ± 1.38	99.07 ± 1.26

Supplementary Table 3. The organ level errors for SSV, MSV and DL approaches in each fold. The reported values represent the mean ± SD. For bladder, the values are not reported in fold 1, as the whole bladder was not included in the scan field-of-view for these patients.

		Error (%)			Absolute percent error (%)		
		**SSV**	**MSV**	**DL**	**SSV**	**MSV**	**DL**
Lesions	Fold 1	0.87 ± 3.92	0.05 ± 3.62	0.27 ± 3.64	1.8 ± 3.6	1.4 ± 3.34	1.51 ± 3.32
	Fold 2	-0.09 ± 1.92	-0.11 ± 1.75	0.05 ± 1.65	0.77± 1.75	0.71 ± 1.6	0.71 ± 1.5
	Fold 3	-0.14 ± 3.44	-0.07 ± 2.3	-0.02 ± 2.03	1.54 ± 3.07	1.14 ± 2	1.03± 1.75
	Fold 4	0.21 ± 1.78	0.09 ± 1.61	0.07 ± 1.68	0.75 ± 1.63	0.63 ± 1.5	0.66 ± 1.54
	Fold 5	-0.19 ± 4.35	-0.46 ± 4.00	-0.01 ± 3.76	2.03 ± 3.85	1.74 ± 3.63	1.64± 3.38
Left Kidney	Fold 1	0.0087 ± 0.22	-0.0058 ± 0.22	0.18 ± 0.27	0.13 ± 0.17	0.12 ± 0.18	0.28± 0.15
	Fold 2	0.12 ± 0.21	0.1 ± 0.21	0.28 ± 0.25	0.2± 0.14	0.18 ± 0.13	0.3 ± 0.22
	Fold 3	0.2 ± 0.12	0.19 ± 0.12	0.13 ± 0.09	0.2 ± 0.12	0.19 ± 0.12	0.14 ± 0.07
	Fold 4	0.1± 0.18	0.08 ± 0.18	0.15 ± 0.2	0.16 ± 0.11	0.16 ± 0.11	0.21 ± 0.13
	Fold 5	0.11 ± 0.12	0.09 ± 0.12	0.29 ± 0.15	0.16 ± 0.05	0.14 ± 0.05	0.3 ± 0.11
Right Kidney	Fold 1	0.12 ± 0.12	0.11 ± 0.12	0.29± 0.15	0.13 ± 0.1	0.13 ± 0.1	0.29 ± 0.15
	Fold 2	0.13 ± 0.12	0.1 ± 0.11	0.27 ± 0.16	0.15 ± 0.08	0.13 ± 0.08	0.27 ± 0.16
	Fold 3	0.22 ± 0.3	0.21 ± 0.3	0.17 ± 0.28	0.25± 0.28	0.24 ± 0.27	0.2 ± 0.25
	Fold 4	-1.25 ± 4.29	-0.82 ± 2.87	-1 ± 3.64	1.49 ± 4.2	1.03 ± 2.8	1.31 ± 3.53
	Fold 5	0.18 ± 0.18	0.16 ± 0.17	0.35 ± 0.18	0.21 ± 0.14	0.19 ± 0.13	0.35 ± 0.18
Liver	Fold 1	0.6 ± 0.43	0.54 ± 0.37	0.8 ± 0.4	0.6 ± 0.43	0.54 ± 0.37	0.79 ± 0.4
	Fold 2	1.58 ± 1.64	1.48 ± 1.51	0.84 ± 0.35	1.58 ± 1.64	1.48 ± 1.51	0.84 ± 0.35
	Fold 3	0.9 ± 0.8	0.85 ± 0.71	0.13 ± 0.33	0.92 ± 0.75	0.85 ± 0.71	0.25 ± 0.25
	Fold 4	0.6 ± 0.27	0.56 ± 0.25	0.4 ± 0.18	0.6± 0.27	0.56± 0.25	0.4 ± 0.18
	Fold 5	0.76 ± 0.64	0.71 ± 0.61	0.81 ± 0.36	0.76 ± 0.64	0.71 ± 0.61	0.81 ± 0.36
Spleen	Fold 1	0.37 ± 0.96	0.35 ± 0.93	0.23 ± 0.38	0.51 ± 0.88	0.5± 0.85	0.33 ± 0.28
	Fold 2	1.85 ± 3.87	1.74 ± 3.68	0.23 ± 0.27	1.85 ± 3.87	1.75 ± 3.68	0.27 ± 0.22
	Fold 3	1.2 ± 1.52	1.13 ± 1.46	-0.02 ± 0.09	1.2 ± 1.52	1.13 ± 1.46	0.06 ± 0.07
	Fold 4	1.35 ± 2.06	1.29 ± 2	0.01 ± 0.18	1.48 ± 1.96	1.43 ± 1.9	0.14 ± 0.09
	Fold 5	0.35± 0.65	0.32± 0.6	0.27 ± 0.2	0.4 ± 0.62	0.37 ± 0.57	0.28 ± 0.18
Bones	Fold 1	-19 ± 8.32	-13.3 ± 5.93	-6.38 ± 5.63	19 ± 8.32	13.3 ± 5.9363	6.38 ± 5.63
	Fold 2	-18.16 ± 13.6	-12.11± 9.33	-8.32 ± 8.54	18.16 ± 13.6	12.11± 9.33	8.32 ± 8.54
	Fold 3	-16.20 ± 8.51	-10.37 ± 5.7	-10.02 ± 5.36	16.2 ± 8.51	10.37 ± 5.7	10.02 ± 5.36
	Fold 4	-16.54 ± 8.47	-11 ± 5.71	-9.62 ± 5.07	16.54 ± 8.47	11 ± 5.71	9.62 ± 5.07
	Fold 5	-15.64 ± 8	-10.78 ± 5.43	-6.5± 4.38	15.64 ± 8	10.78 ± 5.43	6.5 ± 4.38
Lungs	Fold 1	39.88 ± 32.76	4.3 ± 4.72	1.76 ± 3.97	39.88± 32.76	4.3 ± 4.72	2.42 ± 3.56
	Fold 2	44.8 ± 26	5.65 ± 5.3	1.75 ± 1.9	44.8 ± 26	5.74 ± 5.2	2.14 ± 1.4
	Fold 3	56.06 ± 52.63	5.54 ± 6.21	0.48 ± 3.77	56.06 ± 52.63	5.54 ± 6.21	2.57± 2.67
	Fold 4	40.9 ± 49.86	4.15 ± 8.71	-1.35 ± 3.07	40.9 ± 49.86	4.92 ± 8.25	2.35 ± 2.3
	Fold 5	42.77 ± 32.86	3.19 ± 4.02	-0.03 ± 4.16	42.77 ± 32.86	3.64 ± 3.57	3.45 ± 2
Spinal Cord	Fold 1	-8.17 ± 22.21	-8.24 ± 22.11	-7.98 ± 20.62	17.44 ± 14.9	17.23 ± 15.07	11.84 ± 18.38
	Fold 2	9.13 ± 14.81	9.53 ± 14.73	-2.8± 9.36	9.13 ± 14.81	9.53 ± 14.73	5.78± 7.7
	Fold 3	2.77 ± 11.68	4.28 ± 8.55	-9.85 ± 27.68	8.43 ± 8	7.25 ± 5.84	11.72 ± 26.83

	Fold 4	7.51 ± 12.33	7.7 ± 12.32	-1.92 ± 6.27	9.31± 10.87	9.36 ± 10.95	3.44 ± 5.5
	Fold 5	2.64 ± 9.3	2.96 ± 9.14	-1.74 ± 9.18	7.33 ± 5.87	7.35± 5.76	6.07 ± 6.83
Bladder	Fold 1	-	-	-	-	-	-
	Fold 2	0.08 ± 0.11	0.07 ± 0.09	0.27 ± 0.18	0.08 ± 0.11	0.06 ± 0.09	0.27± 0.18
	Fold 3	-0.63± 1.58	-0.64 ± 1.58	-1.34± 1.87	0.92 ± 1.4	0.92± 1.4	1.34 ± 1.87
	Fold 4	-0.09± 0.3	-0.09 ± 0.3	-0.11 ± 0.33	0.22 ± 0.16	0.22 ± 0.16	0.24 ± 0.2
	Fold 5	-6.16 ± 13.7	-6.26 ± 13.63	-1.15 ± 3.2	7.2± 13.01	7.11 ± 13.06	1.8 ± 2.76

Supplementary Table 4. Voxel- and lesion-wise errors for the evaluated approaches resulting in unfavorable results (RE: relative error, RAE: relative absolute error).

Model	Inputs	fold	Voxel wise RE (%)	Voxel wise RAE (%)	Lesions Error (%)	Lesions Absolute Error (%)
Multiple variants (best achieved results)	CT+TIA	0	-14.59 ± 3.85	16.92 ± 3.72	-10.45 ± 7.61	12.21 ± 4.16
		1	-27.72 ± 4.91	28.54 ± 4.84	-19.51 ± 4.68	19.51 ± 4.68
		2	-6.42 ± 6.06	12.68 ± 3.51	-6.20 ± 11.29	12.06 ± 4.40
		3	-32.24 ± 24.18	32.93 ± 23.91	-16.57 ± 17.92	17.31 ± 17.19
		4	-23.17 ± 3.38	27.80 ± 10.78	-11.06 ± 12.22	13.32 ± 9.68
		Overall	-20.96 ± 14.64	23.91 ± 14.07	-12.40 ± 12.10	14.77 ± 9.05
Multiple variants (best achieved results)	CT+MSV	0	-3.24 ± 1.31	4.98 ± 1.13	-4.35 ± 3.20	4.66 ± 2.72
		1	-3.41 ± 1.10	5.00 ± 0.74	-4.16 ± 2.17	4.47 ± 1.41
		2	-2.88 ± 1.17	4.82 ± 0.91	-0.78 ± 4.02	3.05 ± 2.72
		3	-10.71 ± 1.27	11.43 ± 1.24	-4.08 ± 2.83	4.69 ± 1.58
		4	-10.34 ± 1.65	10.71 ± 1.70	-3.99 ± 2.50	4.24 ± 2.04
		Overall	-6.18 ± 3.87	7.44 ± 3.26	-3.31 ± 3.33	4.12 ± 2.25

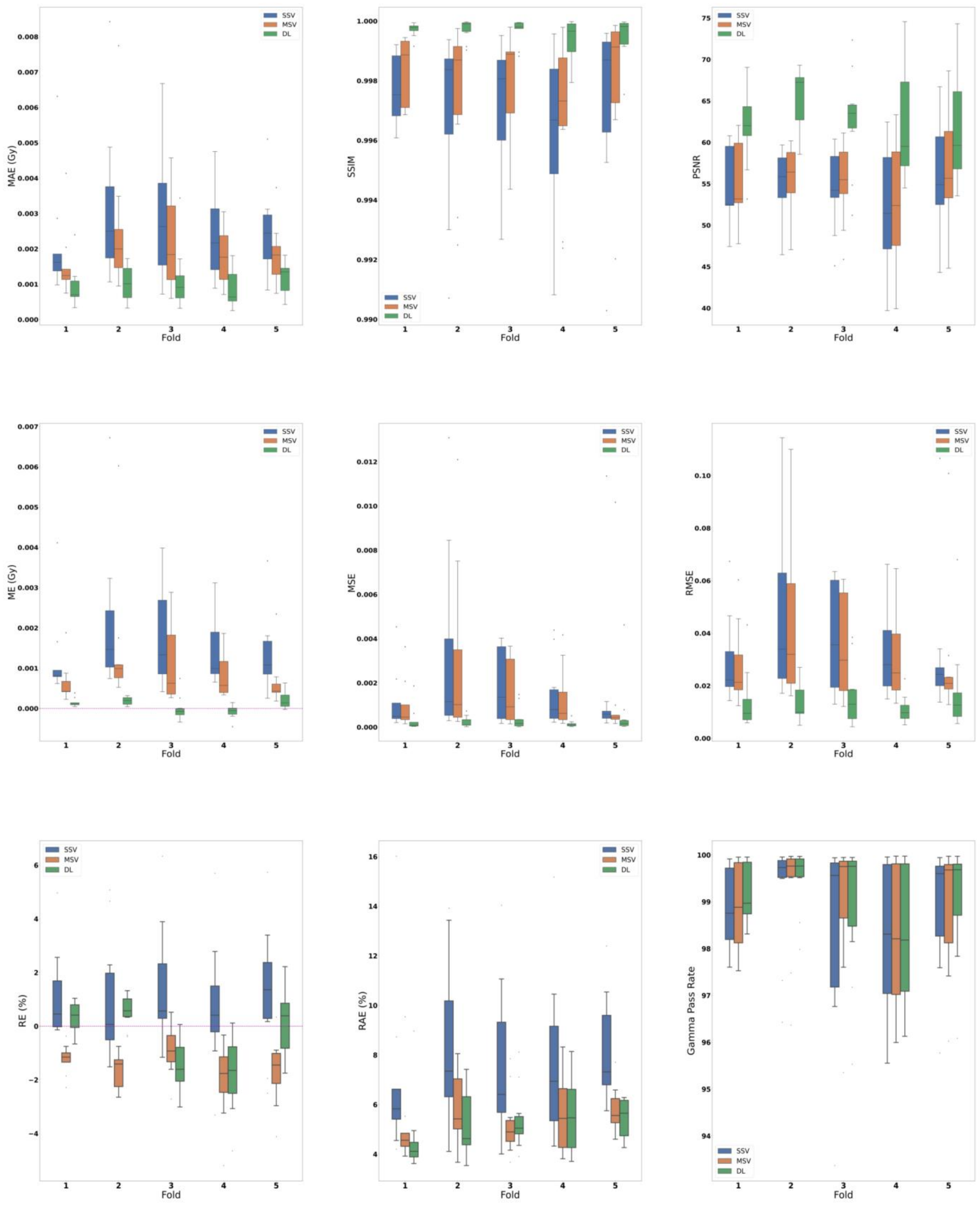

Supplementary figure 1. voxel-wise quantitative metrics of every fold for SSV, MSV, DL approaches.

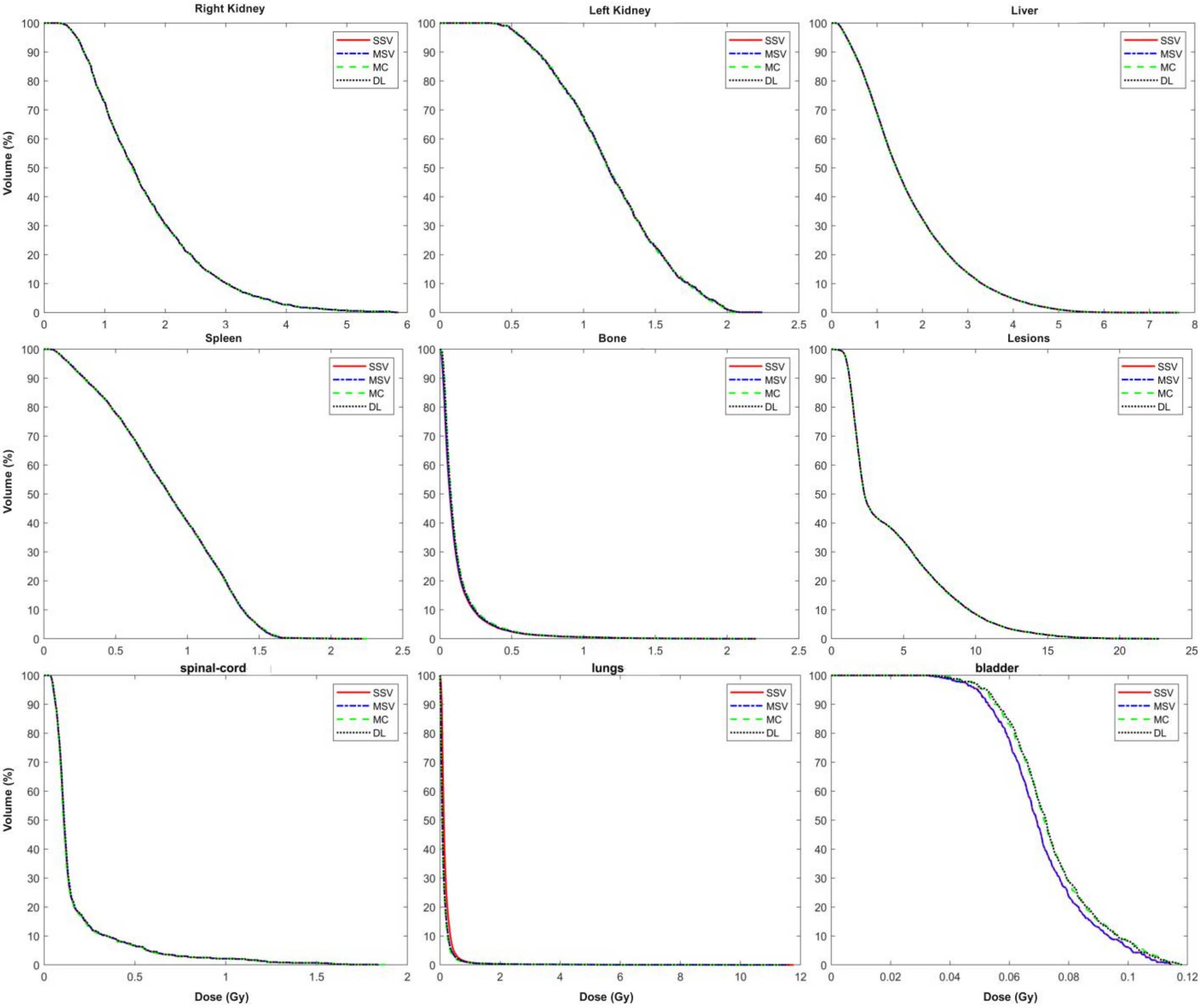

Supplementary figure 2. Representative organ-specific DVHs for all dosimetric approaches.
